# Physicochemical Characterization of Dembi Reservoir Water for Suitability of Fish Production, Southwest Ethiopia

**DOI:** 10.1155/2022/1343044

**Published:** 2022-12-19

**Authors:** Ephrem Chekole, Henok Kassa, Abebe Aschalew, Lalit Ingale

**Affiliations:** ^1^Department of General Forestry, Mizan-Tepi University, P.O. Box 260, Mizan-Tepi, Ethiopia; ^2^Department of Natural Resource Management, Mizan-Tepi University, P.O. Box 260, Mizan-Tepi, Ethiopia; ^3^School of Natural Resources Management and Environmental Sciences, Bahir Dar University, P.O. Box 79, Bahir Dar, Ethiopia

## Abstract

Reservoir water physicochemical characteristics provide important information about water suitability for fish production. Accordingly, the study aimed to characterize the physicochemical characteristics of Dembi reservoir water for sustainable fish production. The study was conducted in Dembi reservoir during the dry season. Water samples were collected in triplicate from selected 10 sampling sites of the reservoir water using manually prepared water sampler made from polyvinyl chloride (PVC) tube. The depth integrated sampling technique was employed to take water samples for all physicochemical characteristics analysis. From the selected 14 physicochemical characteristics, four (temperature, electrical conductivity, pH, and dissolved oxygen) were tested onsite using a multisystem HQ4d electronic meter (probe), whereas the rest 10 water quality characteristics were tested in the laboratory. The result showed that the current average depth of the dam was 5.6 ± 1.61 m. The overall mean values of the water quality characteristics at different sites of the reservoir were as follows: turbidity (26.4 ± 0.44 FTU), total hardness (22.2 ± 0.51 mgL^−1^), NO_3_ (5.4 ± 0.48 mgL^−1^), NO_2_ (0.3 ± 0.11 mgL^−1^), NH_4_ (2.1 ± 0.06 mgL^−1^), PO_4_^−3^ (1.7 ± 0.27 mgL^−1^), total alkalinity (52.5 ± 0.91 mgL^−1^), and BOD_5_ (2.7 ± 0.24 mgL^−1^). There was a significant difference (*p* < 0.05) in all physicochemical characteristics among 10 sampling sites of the reservoir water. The recorded values of all physicochemical characteristics, except NO_2_, NH_4_, and PO_4_^−3^, were found within the recommended standard limit for fish production. The change in reservoir water depth and increase in nutrients shows the presence of sediment siltation and nutrient enrichment. Therefore, proper watershed management practices and waste management should be carried out for sustainable water quality maintenance and fish production.

## 1. Introduction

Fish is one of the major nutritional and commercially important foods in the world. It is a source of nutrition for 4.3 billion people worldwide with 15–20% of their intake of animal protein and in some countries over 50%, and the demand for fish and fishery products is predicted to remain increase because of population growth, economic development, and changes in food habits [1]. However, sustainable fish production is highly dependent on water quality and availability [2].

Water is an essential life-supporting medium for fish and other aquatic organisms. It essentially provides all fish needs, such as food, oxygen, and other helpful environments for breathing, feeding, reproduction, and growth of fishes [3]. The water quality parameters such as water temperature, turbidity, watercolor, dissolved oxygen, BOD, CO_2_, pH, alkalinity, .hardness, calcium, ammonia, nitrite, nitrate, phosphorus, H_2_S, primary productivity, and plankton are essential factors to be considered when planning for high fish production [4].

Dembi hydroelectric power plant was initially constructed for electric power production and had the capacity to provide 800 KW of electric power, however, decommissioned due to the reduction of the water storage capacity of the reservoir because of siltation and electric power operation from the national electric power station (Jimma-Bonga-Teppi) [5]. In addition to generating electricity, the fry fish of Nile tilapia (*Oreochromis niloticus* Linnaeus, 1758) and Redbelly tilapia (*Coptodon Zilli* (Gervais, 1848)) has been introduced in the dam since 1995, aimed to supply fish for the surrounding community [5].

Dembi reservoir is estimated to have an area of 72 km^2^ and a fishery potential of about 383 tons/year [6]. However, the current production of fish from the reservoir is much lower, and even the fish are very small in size. Despite, the southwestern highlands of Ethiopia are the source of water for major rivers and reservoirs, yet, human activities such as agriculture expansion, population increment, and urbanization have led to considerable environmental alteration [7, 8]. For example, the Bench Maji zone natural forest including the upper part of the Dembi reservoir forest reduced from 20% in 1986 to 12% by 2001 [9]. Following agriculture and settlement expansion, surface water decreases in stream base flow and dissolved oxygen (DO) concentration [10, 11]; and increases in nutrient and sediment concentration, periphyton production, and water temperature [12–14]. Furthermore, several wet coffee processing stations have been installed and operational on the upper part of the reservoir. In wet coffee processing stations, coffee by-products (coffee pulp and effluents) are disposed of on the river, thereby, causing water pollution such as high TSS, total ammonia, total nitrogen, COD, BOD_5_, and pH [15]. Similarly, pollutants from urban nonpoint sources add a significant amount of phosphorus and nitrogen to surface water [16].

Knowledge of seasonal reservoir water quality is important for sustainable fish production and management decisions [17]. According to Makinde et al. [18] and Tsegay and Zebib [13], seasonal difference plays a significant role in the reservoir water quality characteristics. In this regard, Gebremichael and Fentahun [19] evaluated selected reservoir water quality properties (temperature, pH, turbidity, nitrate, calcium hardness, conductivity, and total hardness) of the Dembi reservoir during the wet season of 2017. However, no study has been undertaken on reservoir water quality during the dry season. In addition, variability in the spatial distribution of nutrients and sediment determines the reservoir water quality characteristics [20].

Therefore, this study is important to increase our insight on dry season reservoir water quality and the spatial extent of reservoir water quality characteristics. In addition, it provides baseline information for further monitoring and tracking changes in the water quality and maintaining the quality of the reservoir water for sustainable fish production. Thus, the study aimed to determine the physicochemical characteristics of Dembi reservoir water for suitability of fish production during the dry season, with the following specific objectives:

- To determine the level of selected physicochemical characteristics of Dembi reservoir water
- To determine the spatial water quality variation inside the reservoir
- To compare the current water quality of the reservoir with the recommended water quality characteristics for productive fisheries

## 2. Materials and Methods

### 2.1. Description of the Study Area

**Figure 1:**
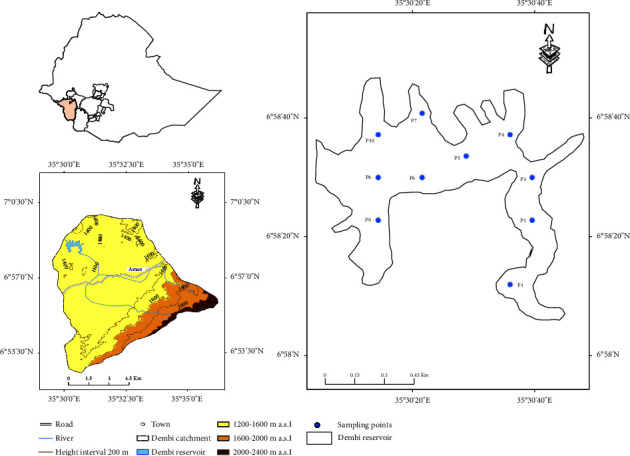
Location of the study site.

The study was conducted in Dembi reservoir, the upper reaches of the White Nile basin. The reservoir is located at about 14 km from Mizan Teferi, the capital of Bench Sheko Zone, and 599 km from Addis Ababa, the capital of Ethiopia. Dembi reservoir is located at 6° 56'–7° 0'N latitude and 35° 30'–35° 36'E longitude. The reservoir is agroecologically located at the lowland elevation, 1440 m a.s.l (Figure 1). The average yearly rainfall in Aman near Mizan Teferi, the main town of Bench Maji Zone, is 1603 (±404) mm y^−1^ [21]. The average air temperature ranges from 13 to 27°C.

### 2.2. Water Sampling Techniques

The water samples were taken during the main dry season (February) in 2019. A preliminary field visit was made using a topographic map and Garmin GPS (model 76CSX ) to fully understand the land features around the reservoir, water flow pattern and shape of reservoir for locating the study area's representative water sampling points. The depth of the reservoir water was measured by a water pipe having a 15 m height with a gauging number marked outside. A total of sixty (60) sampling points were measured to identify the depth contour of the reservoir. A total of ten stations were selected for water quality assessment at the Dembi reservoir (Figure 1).

The depth integrated sampling technique was employed to take water samples for all physicochemical analyses using 600 ml plastic bottles. The bottles were thoroughly washed with distilled water and rinsed repeatedly with water to be sampled. Triplicate water samples from each sampling site were taken in triangle form. The water samples were collected by a manually prepared water sampler from a polyvinylchloride tube (PVC), having 10 m in height and 0.178 m in diameter. Height gauging marks were written on the PVC, and the water sample was collected using gauged PVC tube from the sampling points at 1 m intervals. Each 1 m PVC tube height has a separated internal tube compartment and an inlet hole at the top for the water entrance.

All bottled water samples were capped immediately, stored in an icebox, and transported immediately to Sebeta National Fisheries and Other Aquatic Life Research Center and Haramaya University' Chemistry Laboratory. To avoid decomposition, water samples were immediately filtered in the laboratory using a water jet vacuum pump at low pressure before nutrient analysis.

### 2.3. Physicochemical Analysis of Water

#### 2.3.1. In Situ Analysis

Reservoir water' physicochemical characteristics such as pH, temperature, conductivity, and dissolved oxygen (DO) were tested onsite by using a multisystem HQ4d electronic meter (probe).

#### 2.3.2. Ex Situ (Laboratory) Analysis

The laboratory analysis of physicochemical characteristics of water samples was performed at Sebeta National Fisheries and Other Aquatic Life Research Center and Haramaya University, following the standard methods for the examination of water and wastewater [22, 23]. The reservoir water turbidity was determined by using a digital turbidity meter (formazin turbidimeter), total hardness by the titration method, NO_3_-N by the sodium-salicylate method, NO_2_-N by the colorimetric method, NH_4_-N by the indophenol blue method, total phosphorus (P) by first digesting the unfiltered sample using potassium-peroxodisulphate oxidation, PO_4_^−3^ − P by the ammonium molybdate method, total alkalinity by the titration method, calcium (Ca^2+^) by flame atomic absorption spectrometry (AAS), and biological oxygen demand (BOD) by Azide modification of Winkler's titrimetric method by determining dissolved oxygen contents of the samples before (D1) and after five days (D2) of incubation at 20°C.

#### 2.3.3. Data Analysis

The data obtained from the study were managed in Microsoft Excel. The sources of samples were categorized as inlet, open, and outlet sites to enable analysis. The difference in water physicochemical characteristics between the reservoir sites was tested by one-way ANOVA using SPSS (software version 22), and the mean difference was compared by the least significant difference (LSD) at a 5% level of significance. Moreover, an overall mean value of the physicochemical characteristics of reservoir water was compared with standard values for productive fisheries.

## 3. Results and Discussion

### 3.1. Physical Properties of Dembi Reservoir Water

#### 3.1.1. Depth

The high depth of the reservoir was recorded at *P*6, *P*5, *P*8, *P*7, and *P*4, with an overall mean depth of 5.6 ± 1.61 m (Table 1). There was a significant difference in the reservoir water depth between the 10 sampling stations (*p* < 0.01). It was 17 m during the reservoir construction time since (1991) [24] and has shown a significant reduction from 17 m to 5.6 m in between 28 years. This may be due to the reservoir sediment siltation because of soil erosion from the upper catchment agriculture, grazing, and settlement lands. Similarly, Vacher and Quinn [25] reported that human disturbance on the upper catchment can cause a dramatic change in water depth level.

#### 3.1.2. Turbidity

High reservior water turbidity was recorded at *P*4, *P*1, and *P*3, with an overall mean value of 26.38 ± 0.44 FTU (Table 1). There was a significant difference (*p* < 0.05) in water turbidity level between the different sampling stations of the reservoir water. Since those sites (*P*4, *P*1, and *P*3) were located on an inlet site of the reservoir, the presence of higher turbidity may be due to the presence of river-transported and accumulated suspended sediments and low dilution effect. Similarly, Abate et al. [26] reported higher turbidity in a site near and around the inlet of Lake Hawassa.

The overall mean value of turbidity was found within the recommended range of 20–30 FTU for productive fisheries by Zweigh [27]. Furthermore, it was found in line with the turbidity value at Bira dam (24.3 FTU) by Tessema et al. [28] and Dembi reservoir (wet season) (24.54 ± 3.02 FTU) by Gebremichael and Fentahun [19], higher than in Lake Hawassa (8.44 FTU) by Abate et al. [26], and lower than the turbidity value in Gilgel Gibe reservoir (63.1 FTU) by Woldeab et al. [29].

#### 3.1.3. Temperature

High reservoir water temperature was recorded at *P*4, *P*1, *P*2, *P*3, and *P*10, with an overall mean value of 26.74 ± 0.36°C (Table 1). There was a significant difference (*p* < 0.05) in the level of water temperature among different sampling stations. The presence of high temprature on inlet and outlet sites (*P*4, *P*1, *P*2, *P*3, and *P*10) may be associated with relatively higher turbidity and low water depth on those sites. High water turbidity increases water temperature due to the trapping of heat by turbid water and suspended sediments. Similarly, Tilahun and Ayale [30] observed higher water temperatures in the shallow depth of water at the Selameko reservoir.

The overall mean temperature value was found within the recommended limit for productive fishery by FAO [31] and Bhatnagar and Devi [4], 20–30°C. Furthermore, it was found higher than the temperature in Bira dam (24.16°C) by Tessema et al. [28], Lake Hawassa (21.23°C) by Abate et al. [26], Gilgel Gibe reservoir (23.75°C) by Woldeab et al. [29], and Dembi reservoir (wet season) (25.14 ± 1.12°C) by Gebremichael and Fentahun [19].

#### 3.1.4. Electrical Conductivity

High reservoir water conductivity was recorded at *P*1, *P*2, *P*10, and *P*3, with an overall mean value of 51.03 ± 1.14 *μ*Scm^−1^ (Table 1). There was a significant difference (*p* < 0.05) in water conductivity value among the reservoir water sampling stations. The higher conductivity value recorded at *P*1, *P*2, *P*3, and *P*10 may be due to an increase in the concentration of salts and ions. Similarly, Woldeab et al. [29] reported a higher level of EC in a site near and around the inlet of the Gilgel Gibe reservoir.

The overall mean reservoir water conductivity was found within the recommended limit, 30–5,000 *μ*Scm^−1^, for productive fisheries by Stone and Thomforde [32]. Furthermore, it was found in line with the Dembi reservoir (wet season) EC value (48.94 ± 1.55 *μ*Scm^−1^) by Gebremichael and Fentahun [19]. Conversely, it was lower than the EC value in Bira dam (397.9 *μ*Scm^−1^) by Tessema et al. [28].

### 3.2. Chemical Properties of Dembi Reservoir Water

#### 3.2.1. pH

The reservoir water high pH value was recorded at *P*8, *P*5, *P*7, *P*4, and *P*6, with an overall mean value of 7.7 ± 0.07 (Table 2). There was a significant difference (*p* < 0.05) in water pH value among the different sampling sites. The presence of higher pH in the middle sites (*P*8, *P*5, *P*7, *P*4, and *P*6) may be related to the presence of low nitrate and high water depth on those sites. Higher water depth and volume improve the dilution and buffering capacity, which in turn increased the pH value on the middle sites.

The overall mean value of pH was found within the recommended limit, 6.5–9, for productive fisheries by Ali et al. [33], Santhosh and Singh [34], and Bhatnagar and Devi [4]. Furthermore, it was found in line with the pH level at Lake Hawassa (7.54) by Abate et al. [26] and Selameko reservoir (7.51) by Tilahun and Ayale [28], higher than in Bira dam (7) by Tessema et al. [28], and lower than in Dembi reservoir (wet season) (8.25 ± 0.31) by Askale and Tegegn [19].

#### 3.2.2. Dissolved Oxygen (DO)

High dissolved oxygen in the reservoir was recorded at *P*5, *P*9, and *P*8, with an overall mean value of 4.6 ± 0.12 mgL^−1^ (Table 2). There was a significant difference (*p* < 0.05) in dissolved oxygen content among the different sampling sites. The higher DO content was recorded at *P*5, *P*9, and *P*8, this may be due to lower turbidity values and temperature found on those sites, associated with relatively low biological degradation activities. Furthermore, it may be due to east-west moving cool wind-wave action, which might contribute to the increase in oxygen on middle sites. Similarly, Adeniji [35] and Ibe [36] reported that water temperature, water depth, wind action, and amount of biological degradation activities determine the level of dissolved oxygen content.

The overall mean value of dissolved oxygen was found within the recommended limit for productive fisheries (4-5 mgL^−1^) by Rao et al. [37] and (3–5 mgL^−1^) Bhatnagar and Devi [4]. However, it is lower than the DO level in Lake Hawassa (17.9 mgL^−1^) by Abate et al. [26]. In general, the presence of low DO in Dembi reservoir water may be related to the microbial decomposition of organic wastes from agriculture, municipal solid wastes, coffee by-products (coffee pulp and effluent), and dead aquatic vegetation. Similarly, Srivastava et al. [38] reported that decomposing of organic matter, dissolved gases, mineral waste, and agricultural runoff play a great role in decreasing DO content of the reservoir water.

#### 3.2.3. Total Hardness (TH)

High reservoir water total hardness was recorded at *P*1, *P*10, *P*2, and *P*3, with an overall mean value of 22.16 ± 0.51 mgL^−1^ (Table 2). There was a significant difference (*p* < 0.05) in the value of water hardness among different sampling stations. Higher values of water hardness recorded at *P*1, *P*10, *P*2, and *P*3 may be associated with the presence of higher calcium and magnesium content at those sites, which emanated from decomposed organic wastes from agriculture, municipal solid waste, and coffee by-products. Similarly, APHA [23] reported that higher values for calcium are related to organic wastes (sewage) and weathering of Ca-rich rocks from the upper catchment.

The overall mean value of total hardness at the reservoir water was found within the recommended limit for productive fishery (>15 mgL^−1^) by Swingle [39], (>20 mgL^−1^) Swann [45], (>10 mgL^−1^) Stone and Thomforde [30], and (>20 mgL^−1^) Bhatnagar and Devi [4]. Furthermore, it was found higher than the total hardness value in Dembi reservoir (wet season) (0.27 ± 0.03 mgL^−1^) by Gebremichael and Fentahun [19] and lower than in Lake Hawassa (121.9 mgL^−1^) by Abate et al. [26] and Selameko and Gomit reservoirs (56.0 ± 19.6 mgL^−1^ and 100.2 ± 38.2 mgL^−1^, respectively) by Zelalem et al. [41].

#### 3.2.4. Nitrate-Nitrogen

The reservoir water high nitrate content was recorded at *P*1, *P*3, *P*4, and *P*2, with an overall mean value of 5.44 ± 0.48 mgL^−1^ (Table 2). There was a significant difference (*p* < 0.05) in the nitrate content among the different sampling sites. The higher nitrate content recorded at the inlet sites (*P*1, *P*3, *P*4, and *P*2) may be related to the presence and decomposition of inorganic and organic material transported from agricultural land, municipal waste, and coffee processing station. Similarly, Zelalem et al. [46] reported higher levels of nitrate in the inlet site of the Selameko reservoir.

The overall mean value of nitrate was found in line with the recommended limit for productive fisheries (0.1–4 mgL^−1^) by Santhosh and Singh [37] and (0–100 mgL^−1^) Bhatnagar and Devi [4]. Furthermore, it was found in line with the nitrate level in Lake Hawassa (5.3 ± 0.06 mgL^−1^) by Abate et al. [26] and higher than in Selameko and Gomit reservoirs (2.48 ± 1.51 mgL^−1^ and 2.5 ± 1.9 mgL^−1^, respectively) by Zelalem et al. [41] and Dembi reservoir (wet season) (0.07 ± 0.03 mgL^−1^) by Gebremichael and Fentahun [19].

#### 3.2.5. Nitrite-Nitrogen

High reservoir water nitrite content was recorded at *P*1, *P*4, and *P*3, with an overall mean value of 0.26 ± 0.11 mgL^−1^ (Table 2). There was a significant difference (*p* < 0.05) in the nitrite content of the reservoir water among the different sampling sites. The higher nitrite content recorded on those sites (*P*1, *P*4, and *P*3) may be due to the decomposition of organic materials transported from agricultural lands, settlements, and coffee processing stations. Similarly, Abate et al. [26] reported a higher level of nitrite in the inlet side of Lake Hawassa.

The overall mean nitrite value was found to be higher than the recommended limit for productive fisheries (<0.02 mgL^−1^) by Bhatnagar and Devi [4]. Furthermore, it was found in line with the nitrite level in Selameko reservoir (0.11 ± 0.15 mgL^−1^) by Zelalem et al. [46] and higher than in Lake Hawassa (0.04 ± 0.007 mgL^−1^) by Abate et al. [26].

#### 3.2.6. Ammonia (NH_4_-N)

The reservoir water high ammonia content was recorded at *P*1, *P*4, *P*3, and *P*2, with an overall mean value of 2.13 ± 0.06 mgL^−1^ (Table 2). There was a significant difference (*p* < 0.05) in the ammonia content of water among different sampling sites. Since those sites (*P*1, *P*4, *P*3, and *P*2) were found on an inlet site of the reservoir, the presence of higher content may be associated with the presence of decomposed organic material which is transported from cultivated, grazing, and municipal solid waste dumping sites. Similarly, Osman and Kloas [42] reported a higher level of ammonia on the inlet side of the reservoir water.

The overall mean value of ammonia was found to be higher than the recommended limit for productive fisheries by (<0.2 mgL^−1^) Santhosh and Singh [34], Bhatnagar and Singh [43] and Bhatnagar and Devi [4]. Furthermore, it was found higher than the ammonia level in Geray reservoir (0.06 mgL^−1^) by Mohammed et al. [18].

#### 3.2.7. Total Phosphorus (P)

High reservoir water phosphorus content was recorded at *P*1, *P*4, *P*2, *P*3, *P*10, and *P*7, with an overall mean value of 2.97 ± 0.16 mgL^−1^ (Table 2). There was a significant difference (*p* < 0.05) in total phosphorus content of reservoir water among the different sampling sites. Higher disposal of phosphate from domestic sewages and surface runoff from phosphate-containing fertilizers can lead to higher values of orthophosphate and total phosphorous in reservoir water [44]. Hence, the presence of higher total phosphorous content at *P*1, *P*4, *P*2, *P*3, *P*10, and *P*7 may be due to inorganic and organic nutrient accumulation and decomposition in the inlet sites mainly delivered from upper catchment agriculture, settlement, and wet coffee processing station.

The overall mean value of total phosphorus was found within the recommended limit for productive fisheries (0.01–3 mgL^−1^) by Bhatnagar and Devi [4]. Furthermore, it was found higher than the total phosphorus content in Lake Hawassa and Chamo (0.03 mgL^−1^ and 0.18 mgL^−1^, respectively) by Girma and Ahlgren [45].

#### 3.2.8. Soluble Reactive Phosphorus

The reservoir water high phosphate content was recorded at *P*1, *P*3, *P*4, and *P*2, with an overall mean value of 1.67 ± 0.27 mgL^−1^ (Table 2). The phosphate content of the reservoir water showed a significant difference (*p* < 0.05) among the different sampling sites. The presence of higher phosphate content on those sites (*P*1, *P*3, *P*4, and *P*2) may be due to higher disposal of phosphate source materials from domestic sewages and runoff from agriculture land, municipal solid waste, coffee processing stations, and settlement land. Similarly, Abate et al. [26] and Mohammed et al. [18] reported a higher level of phosphate at the inlet site of reservoir water.

The overall mean value of phosphate at the reservoir was found beyond the recommended limit for productive fisheries by Stone and Thomforde [32] 0.06 mgL^−1^. This implies that the reservoir water was highly polluted by phosphate. Furthermore, it was found higher than the phosphate content in Lake Hawassa (1.12 mgL^−1^) by Abate et al. [26] and lower than in Gomit reservoir (1.96 ± 2.54 mgL^−1^) by Zelalem et al. [41].

#### 3.2.9. Total Alkalinity

High reservoir water total alkalinity content was recorded at *P*1, *P*2, and *P*3, with an overall mean value of 52.54 ± 0.91 mgL^−1^ (Table 2). There was a significant difference (*p* < 0.05) in the alkalinity content of reservoir water among different sampling sites. The higher total alkalinity value recorded at *P*1, *P*2, and *P*3 may be associated with the presence of weathered rocks, waste discharge, and microbial decomposition of organic matter on those sites. Similarly, Mohammed et al. [18] reported higher content of alkalinity on an inlet side of the Geray reservoir.

The overall mean value of total alkalinity at the reservoir was found within the recommended limit for productive fisheries (25–100 mgL^−1^) by Bhatnagar and Devi [4]. Furthermore, it was found lower than the total alkalinity value in Gomit reservoir (91.7 ± 42.2 mgL^−1^) and higher than in the Selameko reservoir (44.5 ± 4.17 mgL^−1^) by Zelalem et al. [41].

#### 3.2.10. Calcium

High reservoir water calcium content was recorded at *P*4, *P*1, *P*2, and *P*10, with an overall mean value of 4.24 ± 0.11 mgL^−1^ (Table 2). There was a significant difference (*p* < 0.05) in calcium content at the reservoir water among the different sampling sites. The higher calcium content recorded at *P*4, *P*1, *P*2, and *P*3 may be due to the presence of sewage from the settlement, municipal solid waste, effluents from the coffee processing station, and weathering of calcium-rich materials from the upper catchment.

The overall mean value of calcium at the reservoir was found within the recommended limit for productive fisheries (4–160 mgL^−1^) by Bhatnagar and Devi [4]. Furthermore, it was found higher than the calcium content in Lake Hawassa (2.56 mgL^−1^) by Abate et al. [26] and Dembi reservoir (wet season) (1.57 ± 0.3 mgL^−1^) by Gebremichael and Fentahun [19]. Conversely, it was found lower than the calcium content in river Gudar (68.7 mgL^−1^) by Wakuma [46].

#### 3.2.11. Biological Oxygen Demand (BOD)

The reservoir water high BOD content was recorded at *P*1, *P*4, *P*3, and *P*2, with an overall mean value of 2.68 ± 0.24 mgL^−1^ (Table 2). There was a significant difference (*p* < 0.05) in the level of BOD among different sampling sites. The presence of higher BOD content at *P*1, *P*4, *P*3, and *P*2 may be associated with the presence of higher nitrate and phosphate content on those sites. Similarly, Abate et al. [26] reported a higher level of BOD on the inlet site of Lake Hawassa.

The overall mean value of BOD at the reservoir was found within the recommended limit for productive fisheries (<10 mgL^−1^) by Santhosh and Singh [34] and (3–6 mgL^−1^) Bhatnagar and Devi [4]. Furthermore, it was found in line with the BOD content in Gilgel Gibe reservoir (2.56 mgL^−1^) by Bizuneh et al. [29] and lower than the BOD content in Lake Hawassa (117 mgL^−1^) by Abate et al. [26].

## 4. Conclusion and Recommendation

Water quality is the most important factor affecting fish health, growth, and production. The reservoir water physicochemical characteristics are spatially variable in Dembi reservoir. Most importantly, Dembi reservoir water quality was suitable for fish production, as confirmed by most of the physicochemical characteristics of the reservoir water. However, the concentration of NO_2_-N, NH_4_-N, and PO_4_-P may cause stress and unsuitable condition for fish production. A significant reduction in the reservoir water depth from 17 m to 5.6 m in between 28 years period, and the increase in nutrient level implies siltation problem and nutrient enrichment. Therefore, proper watershed management practices and waste management should be carried out for sustainable water quality maintenance and fish production in the reservoir.

## Figures and Tables

**Table 1:** Physical properties of Dembi reservoir water (*n* = 10).

Sampling sites	Physical properties			
	Depth (m)	Turbidity (FTU)	Temperature (°C)	Conductivity (*μ*S/cm)
*P*1	3.09 ± 0.01j	27.01 ± 0.03a	27.13 ± 0.03a	52.87 ± 0.05a
*P*2	4.39 ± 0.01h	26.36 ± 0.04cd	27.08 ± 0.03ab	52.23 ± 0.03b
*P*3	5.21 ± 0.02f	26.54 ± 0.06b	26.99 ± 0.03b	51.75 ± 0.05c
*P*4	6.09 ± 0.01e	27.02 ± 0.03a	27.13 ± 0.06a	51.36 ± 0.02d
*P*5	7.58 ± 0.01b	26.43 ± 0.03bc	26.47 ± 0.06d	50.12 ± 0.03f
*P*6	7.89 ± 0.01a	25.55 ± 0.05f	26.51 ± 0.01d	50.0 ± 0.04g
*P*7	6.69 ± 0.01d	25.82 ± 0.07e	26.77 ± 0.04c	50.18 ± 0.02f
*P*8	7.19 ± 0.01c	26.27 ± 0.04d	26.17 ± 0.04e	49.03 ± 0.07h
*P*9	4.49 ± 0.01g	26.48 ± 0.03b	26.23 ± 0.03e	51.00 ± 0.01e
*P*10	3.79 ± 0.01i	26.3 ± 0.05d	26.97 ± 0.03b	51.77 ± 0.07c
Overall mean ± SD	5.64 ± 1.61	26.38 ± 0.44	26.74 ± 0.36	51.03 ± 1.14

The values are given as mean ± SD. *P*1, *P*2, *P*3, *P*4, *P*5, *P*6, *P*7, *P*8, *P*9, and *P*10, reservoir water sampling sites; SD, standard deviation. Means in columns followed by the same letter(s) are not significantly different at 5% level of significance.

**Table 2:** Chemical characteristics of water at Dembi reservoir (*n* = 10).

Chemical characteristics	Sampling sites										Overall mean ± SD
	*P*1	*P*2	*P*3	*P*4	*P*5	*P*6	*P*7	*P*8	*P*9	*P*10	
pH	7.64 ± 0.02bcd	7.6 ± 0.02cd	7.67 ± 0.04bcd	7.71 ± 0.09abc	7.75 ± 0.03ab	7.7 ± 0.02abcd	7.72 ± 0.02abc	7.81 ± 0.03a	7.63 ± 0.01cd	7.59 ± 0.04d	7.68 ± 0.07
DO	4.53 ± 0.02de	4.39 ± 0.04f	4.61 ± 0.06bcd	4.47 ± 0.03ef	4.77 ± 0.07a	4.56 ± 0.01cde	4.65 ± 0.05bcd	4.67 ± 0.01abc	4.73 ± 0.01ab	4.54 ± 0.04de	4.6 ± 0.12
TH	23.0 ± 0.02a	22.47 ± 0.04c	22.38 ± 0.03cd	22.32 ± 0.02d	21.36 ± 0.05i	21.75 ± 0.07g	21.89 ± 0.04f	21.6 ± 0.02h	22.04 ± 0.03e	22.82 ± 0.01b	22.16 ± 0.51
NO_3_^−^	6.27 ± 0.04a	5.28 ± 0.01c	6.13 ± 0.05b	6.07 ± 0.01b	5.13 ± 0.05d	5.11 ± 0.07de	5.1 ± 0.05de	5.02 ± 0.01e	5.16 ± 0.04d	5.17 ± 0.04d	5.44 ± 0.48
NO_2_	0.47 ± 0.01a	0.29 ± 0.02d	0.34 ± 0.01c	0.4 ± 0.015b	0.21 ± 0.02e	0.21 ± 0.03e	0.13 ± 0.01g	0.17 ± 0.02ef	0.21 ± 0.02e	0.21 ± 0.01e	0.26 ± 0.11
NH_4_	2.23 ± 0.06a	2.15 ± 0.06ab	2.17 ± 0.05ab	2.17 ± 0.05ab	2.09 ± 0.04b	2.13 ± 0.05ab	2.02 ± 0.04ab	2.08 ± 0.04b	2.07 ± 0.04b	2.13 ± 0.05ab	2.13 ± 0.06
TP	3.24 ± 0.04a	3.11 ± 0.02b	3.03 ± 0.02c	3.12 ± 0.03b	2.9 ± 0.05d	2.88 ± 0.02d	3.01 ± 0.01c	2.73 ± 0.03e	2.73 ± 0.04e	3.02 ± 0.01c	2.97 ± 0.16
SRP	2.07 ± 0.01a	1.76 ± 0.01b	2.06 ± 0.01a	2.03 ± 0.02a	1.42 ± 0.05c	1.52 ± 0.06c	1.4 ± 0.03c	1.52 ± 0.06c	1.5 ± 0.06c	1.42 ± 0.05c	1.67 ± 0.27
TA	53.64 ± 0.05a	53.33 ± 0.06b	53.14 ± 0.06c	51.83 ± 0.04e	51.31 ± 0.03f	52.65 ± 0.01d	53.0 ± 0.04c	53.03 ± 0.06c	50.73 ± 0.05g	52.67 ± 0.06d	52.54 ± 0.91
Ca	4.36 ± 0.06ab	4.34 ± 0.01ab	4.23 ± 0.03cd	4.37 ± 0.03a	4.15 ± 0.05de	4.15 ± 0.01de	4.09 ± 0.03e	4.16 ± 0.01de	4.26 ± 0.05bc	4.33 ± 0.03ab	4.24 ± 0.11
BOD	3.04 ± 0.01a	2.7 ± 0.02b	2.97 ± 0.06a	3.02 ± 0.02a	2.58 ± 0.03cd	2.48 ± 0.04d	2.3 ± 0.02e	2.58 ± 0.05c	2.57 ± 0.01cd	2.61 ± 0.03bc	2.68 ± 0.24

The values are given as mean ± SD. Chemical characteristics: DO (mg L^−1^), TH (mg L^−1^), NO_3_^−^N (mg L^−1^), NO_2_-N (mg L^−1^), NH_4_-N (mg L^−1^), TP (mg L^−1^), SRP (mg L^−1^), TA (mg L^−1^), Ca (mg L^−1^), and BOD (mg L^−1^). Mean value of reservoir sampling sites water chemical characteristics with a similar letter in a row are not significantly different from each other at 5% level of significance.

## Data Availability

The data used to support this study are given in Tables 1 and 2.

## Abbreviations

PVC: Polyvinyl chloride
BOD: Biochemical oxygen demand
CO_2_: Carbon dioxide
EEPCo: Ethiopian Electric Power Corporation
DO: Dissolved oxygen
GPS: Global positioning system
APHA: American Public Health Association
AAS: Atomic absorption spectrophotometry
LSD: Least significant difference
FTU: Formazin Turbidity Units
FAO: Food and Agriculture Organization
TH: Total hardness
SRP: Soluble reactive phosphorus
TA: Total alkalinity
SD: Standard deviation.
